# Case Report: Identification of a Novel Heterozygous Missense Mutation in *COL4A3* Gene Causing Variable Phenotypes in an Autosomal-Dominant Alport Syndrome Family

**DOI:** 10.3389/fgene.2022.839212

**Published:** 2022-03-29

**Authors:** Yanglin Hu, Wei Li, Lulu Tian, Shuai Fu, Yonglong Min, Jia Liu, Fei Xiong

**Affiliations:** ^1^ Department of Nephrology, Wuhan No.1 Hospital, Wuhan, China; ^2^ Department of Endocrinology, Wuhan No.1 Hospital, Wuhan, China; ^3^ Department of Blood Purification, Central Theater General Hospital, Wuhan, China

**Keywords:** human genetics, next-generation sequencing, whole-exome sequencing, Alport syndrome, collagen type IV, *COL4A3*, novel variant

## Abstract

Alport syndrome (AS) is a genetic kidney disease of basement membrane collagen disorder accounting for approximately 2% of ESRD patients. Next-generation and whole-exome sequencing methods are increasingly frequently used as an efficient tool not only for the diagnosis of AS but also for the establishment of genotype–phenotype correlation. We herein report the identification of a novel heterozygous missense mutation in *COL4A3* gene (c.G3566A: p.G1189E) causing variable phenotypes in an ADAS Family based on the combination of clinical, histologic, pedigree, and genetic sequencing information. The proband is a 48-year-old Chinese woman suffering from persistent subnephrotic proteinuria and intermittent hematuria without renal function impairment over a 10-year time-span. Renal biopsy showed diffuse thin basement membrane and focal interstitial foam cell infiltration. The proband’s mother progressed to end-stage renal failure and the proband’s sister presented with subnephrotic proteinuria and intermittent hematuria as well. AS was highly suspected and confirmed by exome sequencing which revealed a novel heterozygous missense mutation in *COL4A3* gene (c.G3566A: p.G1189E) in all the affected family members, although their current medical conditions vary significantly. Our present finding emphasizes the significance of next-generation sequencing technology for genetic screening which gives us an accurate clinical diagnosis of ADAS patients. The identification of c.G3566A as a new ADAS-related mutation contributes to both genetic diagnosis of ADAS and further functional study of *COL4A3*. The variable phenotypes from the same genotype of our case also provide more information to genotype–phenotype correlation study.

## Introduction

Alport syndrome (AS) is a genetically and phenotypically heterogeneous disorder of glomerular, cochlear, and ocular basement membranes caused by mutations in the genes *COL4A3*, *COL4A4*, and *COL4A5*, which encode type collagen IVα3, α4, and α5 chains, respectively (Kashtan, 2021). It is the second most common cause of genetic kidney disease after autosomal-dominant polycystic kidney disease (Savige et al., 2021). The collagen α3, 4, and 5 are the major components of the mature glomerular basement membrane (GBM) in the kidney, eye, and cochlea. Disruption or alteration of those collagen leads to breakdown of their structure and function. Various clinical manifestations ranging from isolated, asymptomatic hematuria, through hematuria and proteinuria, to progressive renal disease, sensorineural deafness, lenticonus, and retinal flecks may occur (Kashtan et al., 2018).

The disease is transmitted in an X-linked manner in the case of *COL4A5* mutation, in an autosomal manner when mutations are located in the *COL4A3* or *COL4A4* genes, or in a digenic inheritance manner when a combination of two mutations in different genes occurs (Quinlan and Rheault, 2021). X-linked is the major inheritance pattern which accounts for 70–80% of AS patients (Quinlan and Rheault, 2021). It has been increasingly recognized that autosomal-dominant AS (ADAS) accounts for a larger percentage of patients with AS than previously recognized, up to 19–31% of affected patients, which made it the second most common inheritance pattern of AS (Fallerini et al., 2014; Morinière et al., 2014; Quinlan and Rheault, 2021). Increased application of next-generation technology in the evaluation of familial renal disease has led to the confirmed diagnosis of AS patients, especially ADAS, in the molecular level and reveals its correlation with histology and clinical manifestation (Groopman et al., 2019).

In this study, three members of a Chinese AS family with various phenotypes were identified as having the same novel heterozygous missense mutation, c.G3566A: p.G1189E, in the *COL4A3* gene. The result allows us to conclude an accurate diagnosis of the family. More importantly, it expands the mutational spectrum of *COL4A3* gene associated with ADAS family, confirms that heterozygous mutations in *COL4A3* gene are associated with a spectrum of phenotypes ranging from asymptomatic abnormal urine analysis to progressive renal disease, which provides more information about genotype–phenotype correlation, and also highlights the efficiency of high-throughput next-generation sequencing technology for identifying candidate variants.

## Case Presentation

### Clinical History and Laboratory Data

**FIGURE 1:**
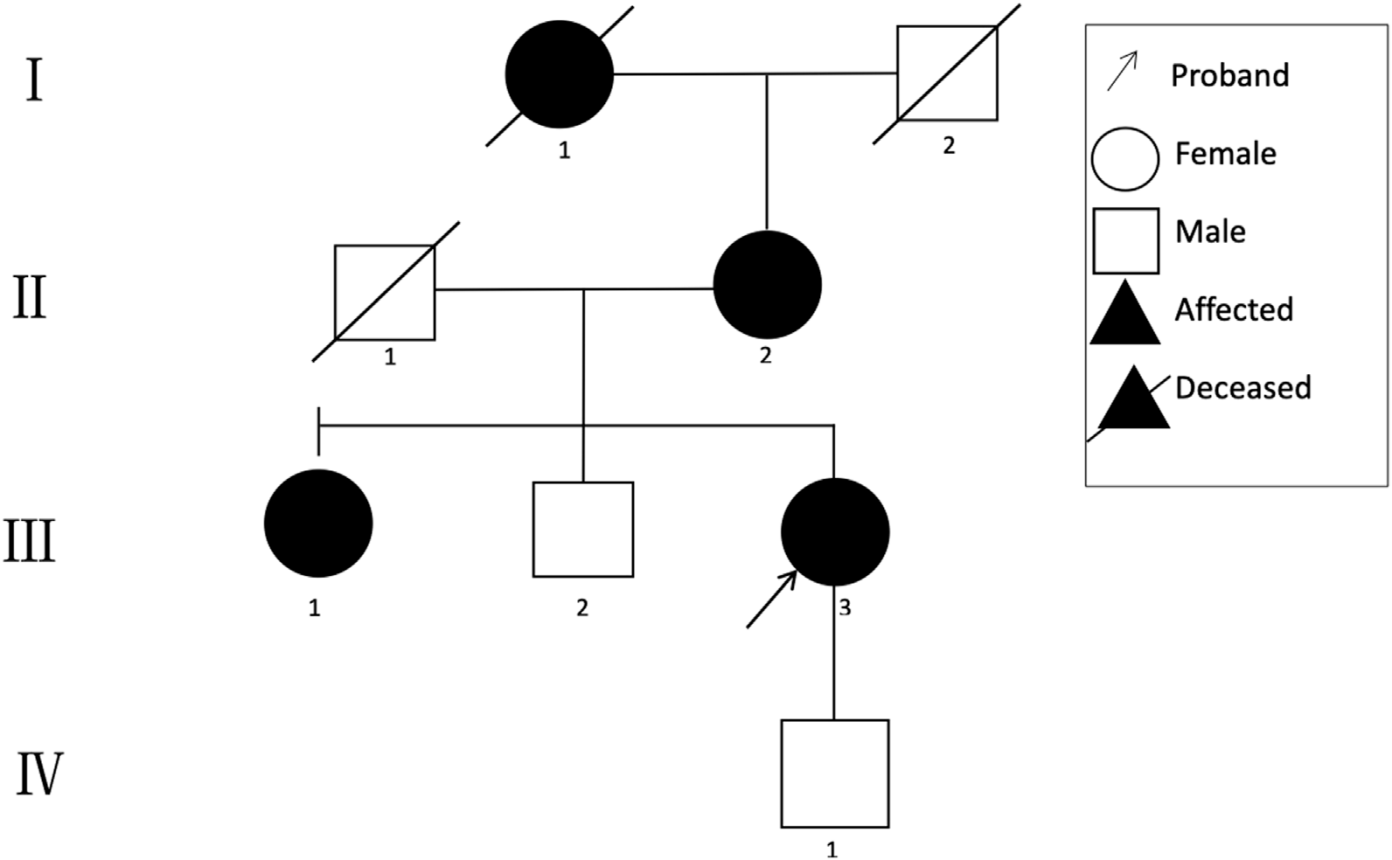
Pedigree of the family.

A 48-year-old Chinese woman was referred to our nephrology department for consultation regarding persistent sub-nephrotic range proteinuria and intermittent hematuria over a 10-year span. Her mother and her elder sister also had history of kidney disease and, possibly, her maternal grandmother as well. She is not from a consanguineous family and was delivered at full term with normal development (Figure 1). 10 years ago, she found herself with binocular edema and the following urine test showed sub-nephrotic–range proteinuria ranging from 1.0 to 2.9 g/g and mild intermittent hematuria with normal serum albumin and creatinine levels. Traditional medical drugs and ACEI (angiotensin-converting enzyme inhibitors)/ARB (angiotensin receptor blocker) were prescribed to her. But there was no significant improvement and she did not take the medications regularly. Renal biopsy was suggested and performed in the year 2018. Only mild mesangial expansion, diffuse thin GBM, and very focal interstitial foam cell infiltration was found. Gene analysis was suggested, but she refused because of financial reasons. Now she agreed to do gene analysis because her urine abnormalities persisted and had the tendency of getting worse, especially proteinuria, and most importantly, her mother reached ESRD and started renal replacement therapy.

Laboratory data when gene analysis was done included UPC of 3.26 g/g, hematuria of + with 70% dysmorphic RBCs, normal serum albumin level 41.1 g/L (34–54 g/L), serum creatinine 62 umol/L (0.7 mg/dl, 44–97 umol/L), and CHOL 7.06 mmol/L (3.38–5.17 mmol/L). Hepatitis C antibody, Hepatitis B surface antigen, HIV, antinuclear antibody (ANA), antineutrophil cytoplasmic antibodies (ANCA), and free light chain were all negative. No high-frequency hearing loss or ocular lesion was found.

The proband’s elder sister (52 years old) also suffered from a similar medical history, but shorter and milder. She presented with persistent sub-nephrotic–range (0.5–2.4 g/g) proteinuria and mild intermittent hematuria for 4 years without kidney failure. She was also treated with ACEI/ARB and traditional Chinese medical drugs, but she was not quite compliant.

The proband’s mother (80 years old) presented with chronic renal failure (CKD stage 4), sub-nephrotic–range proteinuria and intermittent hematuria for 5 years and reached ESRD this year and started with hemodialysis. Her mother had cataract surgery 8 years ago and intermittent tinnitus for a long time.

The proband’s maternal grandmother also may have had proteinuria because of the symptomatic foamy urine, but she had already died and no medical test was done to confirm the suspicion.

The clinical characteristics of the family are summarized in Table 1.

**TABLE 1:** Clinical laboratory and gene data of the family.

Subject	I:1	I:2	II:1	I:2	III:1	III:2	III:3	IV:1
Sex	Female	Male	Male	Female	Female	Male	Female	Male
Age	N/A	N/A	N/A	80	52	55	48	24
Blood pressure	N/A	N/A	N/A	Normal	Normal	Normal	Normal	Normal
Hematuria	N/A	N/A	N/A	Intermittent2+	Intermittent +	Normal	Intermittent+	Normal
Proteinuria	Foamy urine	N/A	N/A	2+–3+	+–2+	Normal	2+	Normal
Alb (g/l)	N/A	N/A	N/A	33.1	43.2	Normal	41.1	Normal
BUN (mmol/l)	N/A	N/A	N/A	24.1	5.8	Normal	6.2	Normal
Scr (mg/dl)	N/A	N/A	N/A	8.19	0.68	Normal	0.7	Normal
Audiological examination	N/A	N/A	N/A	Tinnitus	Normal	Normal	Normal	Normal
Ophthalmic examination	N/A	N/A	N/A	Cataract	Normal	Normal	Normal	Normal
Genotype	N/A	N/A	N/A	Heterozygous	Heterozygous	-	Heterozygous	Wild-type

Alb, serum Albumin; BUN, blood urea nitrogen; Scr, serum creatinine values; N/A, not available; -, no data.

### Light Microscopy Examination

**FIGURE 2:**
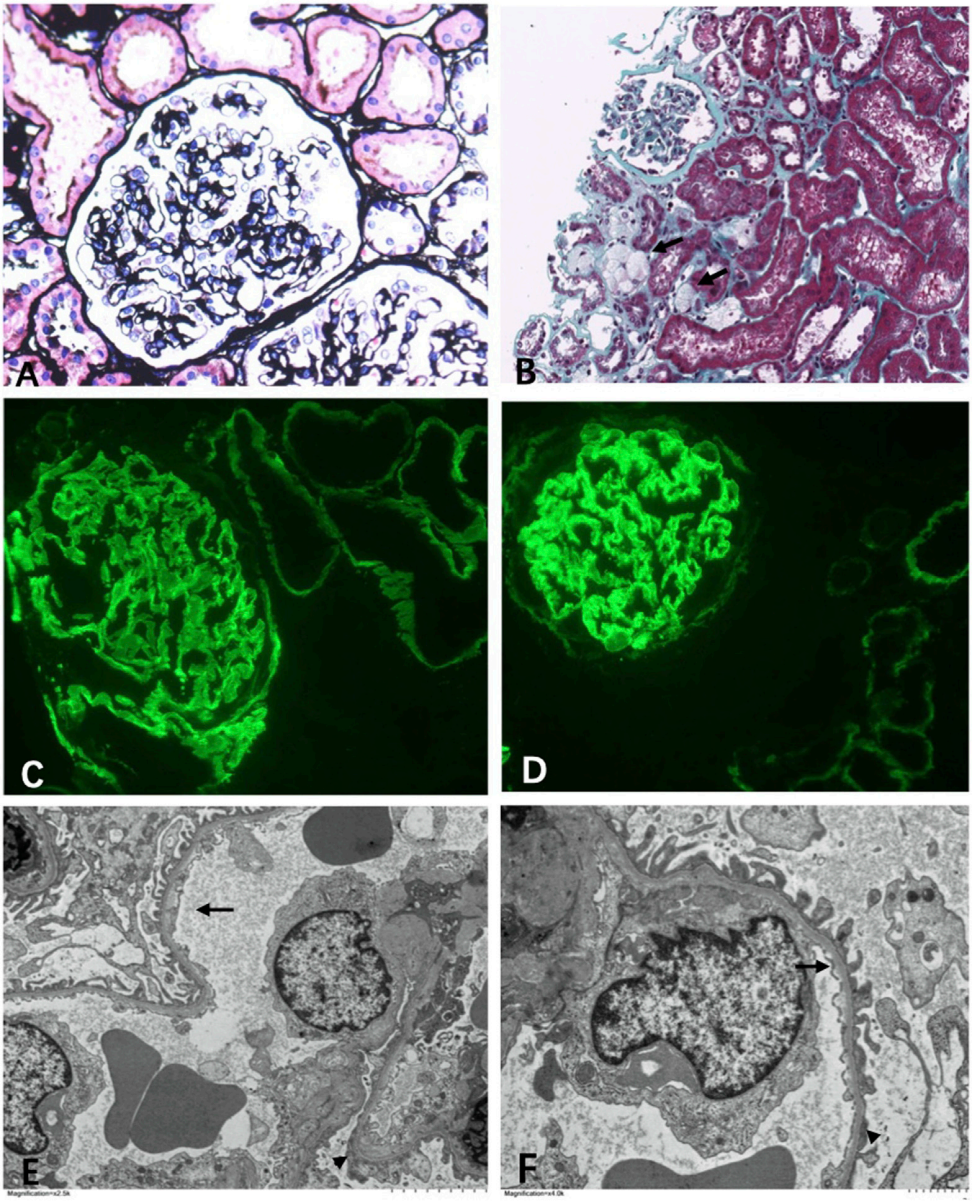
Light microscopy. **(A)** Mild segmental mesangial expansion (Manson’s Trichome stain ×400). **(B)** Focal foam cells infiltration (←) (Jones silver stain ×100). Immunofluorescence. **(C)** α3 collagen IV was well distributed in the kidney (×400). **(D)** α5 collagen IV was well distributed in the kidney (×400). Electromicroscopy. **(E,F)** Diffuse thin basement membrane (←) with segmental overlapping podocyte effacement (▲) (×6000).

In multiple levels of section examined with H&E, PAS, trichrome, silver, and elastic tissue stains, up to 20 glomeruli were noted, 30% of which were globally sclerosed. The remaining glomeruli were slightly enlarged. Mesangial regions were segmentally minimally expanded due to matrix deposition. No endocapillary hypercellularity or crescents were found. Chronic changes were noted in the interstitial compartment with focal mild fibrosis and tubular atrophy. Very focal foam cells were found in the interstitium (Figures 2A,B). Protein cast and RBC cast could be seen. Arterioles and arteries demonstrated mild hyalinosis.

### Immunofluorescence Examination

Immunofluorescence study revealed that the α3 and α5 collagen IV chains were well distributed in the kidney (Figures 2C,D). No diagnostic glomerular or extra-glomerular staining was seen with antisera specific for IgG, IgA, IgM, C3, C1q, fibrin, and kappa and lambda light chains.

### Electron Microscopy Examination

One glomerulus was examined by electron microscopy. The glomerulus showed diffuse thinning of the glomerular capillary membrane without lamination or splitting and with segmental effacement of overlapping foot processes (Figures 2E, F). Endothelial cells were activated. No discrete immune complex electron dense deposits were identified.

### Gene-Panel Design Sequencing and Variant Identification

Peripheral blood (5 ml) from the proband (III:3) was collected in EDTA anticoagulant tubes, and DNA samples were obtained using an extraction kit. Gel electrophoresis was used to identify, quantify, and purify nucleic acid fragments. The amount of DNA was quantified using a Qubit 3.0 fluorometer. DNA was randomly broken into 250–300 bp long fragments using a Covaris S2 Sonicator (Covaris). The DNA sequencing library was prepared including end-repair, A-tailing, and adapter ligation. After pooling with specific indexes, the DNA library samples were hybridized and captured by streptavidin-coated magnetic beads. Quality assessment was processed after PCR amplification. The target region of genetic kidney disease–related genes (including AS) was enriched, and a total exon library was constructed. Targeted sequences and prepared samples were run on a Illumina NovaSeq PE150 high-throughput sequencing instrument. Average sequencing depth of the target area is 156.57 with 99.54% coverage.

### Bioinformatics Analysis

The obtained sequences were compared to those of the human reference genome hg19 (UCSC) using Borrows–Wheeler Aligner software (version 0.7.15 http://bio-bwa.sourceforge.net/) to acquire valid sequences, and variant calling was performed using GATK3.1.1 with HaplotypeCaller. The called variants were annotated using ANNOVAR.

### Sanger Sequencing

To validate putative variants, Sanger sequencing was performed using an ABI 3730XL DNA analyzer. Primers flanking the candidate loci were designed based on the reference genomic sequences of the Human Genome from GenBank in NCBI and synthesized by Invitrogen, Shanghai, China. The heterozygous variants identified through targeted next-generation sequencing were verified through Sanger sequencing using the following primers: F:5-ACACTTTAGAGCTTACACCATGCTT-3, R:5-GGTAACATGAGGAGGAGGTTGAAAA-3.

### Identification of Novel Variants

**FIGURE 3:**
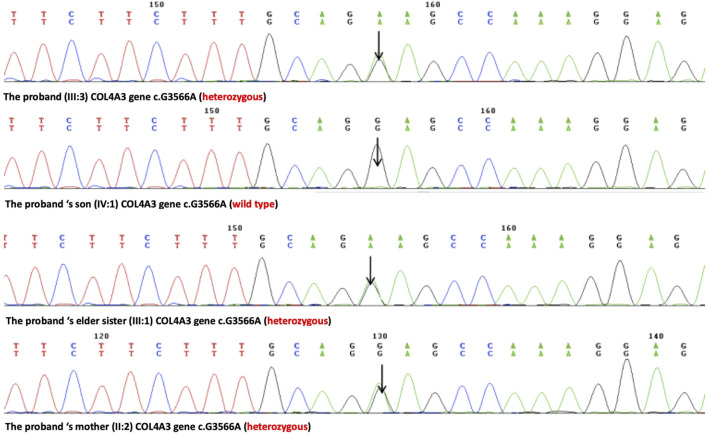
Sanger sequencing results of the c.G3566A (p.G1189E) variant at the 43rd exon of the COL4A3 gene of the proband (III:3), her son (IV:1), her elder sister (III:1), and her mother (II:2).

A novel heterozygous missense mutation, c.G3566A, has been identified in the *COL4A3* gene (chr2:228162390) in the proband (Figure 3). The heterozygous point mutation (c.3566G > A) in exon 42 of *COL4A3* gene causes a codon that codes for a different amino acid which results in a substitution of glycine by glutamic acid at amino acid position 1189 (p.G1189E). It locates in position 1 glycine residues in the Gly-X-Y repeats in the intermediate collagenous domains. The variant was confirmed by Sanger sequencing, and her family members (II:2, III:1, III:3, and IV:1; Figure 3) were subsequently assessed. The c.3566G > A (p.G1189E) *COL4A3* variant was not detected in her son (who was also clinically normal), whereas her mother and her sister were both heterozygous (who were both affected by kidney diseases with different phenotypes) (Figure 3). This variant was not found in GnomAD, ExAC, or in 1000 Genome Databases. The REVEL computational prediction analysis tool produced a score of 0.974. According to the refining criteria of AS variant interpretation guidelines of the American College of Medical Genetics and Genomics (ACMG) (Richards et al., 2015; Savige et al., 2021), this variant is classified as a “Likely pathogenic” (PM1, PM2, PP3, and PP4). Combing through the clinical, pathology, and pedigree data, we came to the conclusion that this is the novel pathogenic variant for this ADAS family.

### Follow Up

Once the diagnosis was made, the patient and her sister were prescribed with ACEI. She and her family were all satisfied because the diagnosis was made and the treatment and prognosis were clear. But unfortunately, she was lost to follow up after the diagnosis.

## Discussion

Six genes (*COL4A1–COL4A6*) are arranged in three pairs (*COL4A1–COL4A2*, *COL4A3–COL4A4*, and *COL4A5–COL4A6*) and locate on chromosomes 13, 2, and X, respectively. They encode six isoforms, α1 to α6 of type IV collagen, which is the major structural component of all basement membranes (Quinlan and Rheault, 2021). Type IV collagen is a heterotrimer composed of three α chains coiled around one another to form a helical protomer (Timpl, 1989; Jennette, 2015; Savige et al., 2021). Three distinct networks have been recognized: α1.α1.α2-α1.α1.α2, α3.α4.α5-α3.α4.α5, and α1.α1.α2-α5.α5.α6 (Abrahamson et al., 2009; Quinlan and Rheault, 2021). In the mature kidney, the α3α4α5 network is the main component of the GBM, encoded by the *COL4A3*, *COL4A4*, and *COL4A5* genes, respectively (Kashtan et al., 2018; Kashtan, 2021; Quinlan and Rheault, 2021; Savige et al., 2021). Disease-causing mutations in these genes will lead to AS with various phenotypes transmitted in different ways. A milder disorder previously known as “benign familial hematuria” or “thin basement membrane nephropathy” has a common molecular basis and potentially may not be “benign.” The entity “benign familial hematuria” or “thin basement membrane nephropathy” has been eliminated by the Classification Working Group in 2018 to avoid the possible harm of classifying a patient with a benign prognosis (Kashtan et al., 2018).

The family we reported here was clinically highly suspected of AS because the proband had hematuria occurring together with a family history of hematuria and renal impairment and an abnormal GBM appearance (Savige et al., 2013). But the intermittent but not persistent hematuria, the increasing proteinuria, no extra-renal symptoms, the foam cells in the kidney biopsy without GBM lamination, the podocyte effacement, and very different clinical phenotypes within the family called for definite gene variant identification to confirm AS diagnosis excluding other hereditary or non-hereditary disease such as podocytopathy, further understanding of phenotype–genotype relationship, and better prediction of prognosis to guide clinical management.

**FIGURE 4:**
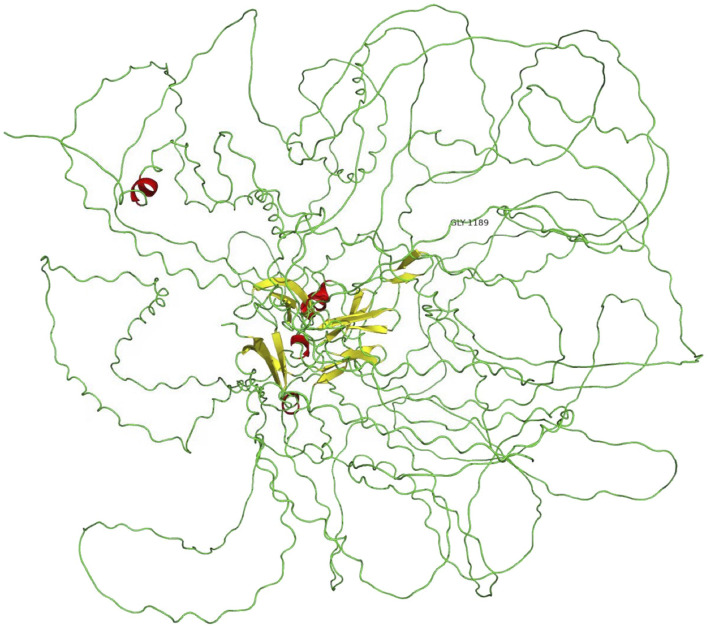
Location of p.G1189E in COL4A3 protein.

A novel heterozygous missense mutation, c.G3566A, was identified by next-generation sequencing, which locates in exon42 of the *COL4A3* gene on chromosome 2 in which G in the 3566rd position was changed to A (c.G3566A), leading to the amino acid change from glycine to glutamic acid (p.G1189E, Figure 4). This variant is absent from all population allele frequency databases (GnomAD, ExAC, and 1000 Genome Databases) and has to our knowledge not been reported in the literature. The normal α3 chain encoded by the *COL4A3* gene is composed of 1,670 amino acids. It has a 28–amino acid leucine-rich signal peptide, followed by a 1,410–amino acid collagenous domain, and a 232–amino acid C-terminal NC1 domain (Mariyama et al., 1994). The collagenous domain begins with a 14–amino acid noncollagenous sequence that includes 4 cysteines, and the collagenous repeat Gly-X-Y is interrupted 23 times by short noncollagenous sequences (Mariyama et al., 1994; Heidet et al., 2001). The collagenous domain is characterized by the repeated Gly-X-Y triplet sequence, in which every third amino acid is a glycine. The presence of glycine is crucial for proper triple-helix formation, because glycine is the only amino acid small enough to fit into the center of the triple helix (Heidet et al., 2001; Jennette, 2015). If glycine is substituted by any other residue, normal folding of the triple-helical collagen molecule would be disrupted and consequently lead to partial or complete absence of these collagens within mature GBM (Yeo et al., 2020). Also, the misfold collagen molecules would also be highly sensitive to proteases and thus prone to degradation (Yeo et al., 2020). The refining ACMG criteria for the molecular diagnostics of AS stated that most glycine residues in the collagenous domain of the collagen IV α5, α3, and α4 chains should be recognized as critical residues equivalent to a functional domain and, therefore, identified position 1 glycine residues in the Gly-X-Y repeats in the intermediate collagenous domains in the collagen IV α5, α3, and α4 chains as “mutational hotspots” (PM1) (Savige et al., 2021). According to the refining criteria, the new variant, c.3566G > A (p.G1189E), locates in the collagenous domain, and PM1 can be applied. The REVEL computational prediction analysis tool produced a score of 0.974, which is above the threshold necessary to apply PP3. All three patients in the family identified with hematuria (PP4). This variant meets criteria to be classified as “likely pathogenic” for AS based on the refining ACMG criteria (Savige et al., 2021). After associating with the clinical, pathology, and pedigree data, we eventually have the final diagnosis of ADAS with this novel heterozygous missense mutation c.G3566A in *COL4A3* gene as the pathogenic variant.

Autosomal-dominant inheritance was mistakenly considered to be very rare previously (Savige et al., 2013; Zhang and Ding, 2018). But as the increasing application of gene sequencing technology, the old narrowed definition of AS was broadened, such as the reclassification of “thin basement membrane nephropathy” and focal segmental glomerulosclerosis with any variant of *COL4A3-5*, as both could be a manifestation of ADAS if the variant located in *COL4A3* and *COL4A4* (Kamiyoshi et al., 2016; Kashtan et al., 2018; Kashtan, 2021; Quinlan and Rheault, 2021; Savige et al., 2021). GBM thinning, which was previously considered to be a very important pathologic finding, can be found in various situations including young male patients with X-linked AS, female patients of any age with X-linked AS, young male and female patients with autosomal recessive AS, and male and female patients with heterozygous mutations in the *COL4A3* and *COL4A4* genes transmitted in an autosomal-dominant pattern who may or may not exhibit progressive renal disease (Kashtan et al., 2018), which is well demonstrated in our case. With a substantial portion of the unrevealed autosomal-dominant inheritance patients with various clinical presentations being identified by NGS (Kamiyoshi et al., 2016; Kashtan et al., 2018; Kashtan, 2021; Quinlan and Rheault, 2021; Savige et al., 2021), ADAS is now considered to account for a larger percentage of patients with AS than previously recognized, which is up to 19–31% (Fallerini et al., 2014; Morinière et al., 2014; Kashtan et al., 2018; Kashtan, 2021; Quinlan and Rheault, 2021).

Accordingly, this makes phenotype–genotype correlation data lag behind for variants in *COL4A3* and *COL4A4* (Rehm et al., 2015). Men with X-linked AS invariably develop kidney failure, and their phenotype is strongly influenced by genotype in that large deletions and nonsense mutations conferred a 90% probability of kidney failure before age 30, compared with a 70% risk with splice site mutations and a 50% risk with missense mutations (Jais et al., 2000; Yamamura et al., 2020). The phenotype of ADAS can vary from isolated hematuria through progressive kidney disease to renal failure, hearing loss, and ocular lesions. What we know now is that renal failure is not common, but recognized increasingly, and hearing loss is unusual, affecting 4–13% of individuals, as are ocular lesions (Kamiyoshi et al., 2016; Kashtan, 2021). But the exact phenotype–genotype correlation has yet to be established. Our case demonstrated that heterozygous missense mutation c.G3566A in *COL4A3* gene can cause various phenotypes in one family. It suggested that the severity of clinical manifestations may not be entirely attributed to the *COL4A3* genetic variant itself in patients. The reasons for this variability of expression/incomplete penetrance are complicated, including modifier genes that ameliorate or exacerbate the effects of mutations on synthesis, assembly, deposition, or function (or a combination of these) of the collagen IV α345 and factors such as smoking, high blood pressure, and dietary levels of salt and animal protein (Kashtan et al., 2018). According to that, an estimated risk of ESRD is given to ADAS patients with 20% or more among those with risk factors, <1% among those without any risk factors including proteinuria, FSGS, GBM thickening and lamination, sensorineural hearing loss, or evidence of progression in patient or family, genetic modifiers (Kashtan et al., 2018). According to the prognosis estimation, the proband and her sister both have more than 20% risk for progression to ESRD because she has the risk factor of proteinuria and family history of ESRD. To delay renal failure, ACEI should be continuously used. Because although there is no curative treatment for AS, early ACEI therapeutic interventions have been shown to be effective both in AS patients and AS animal models, especially if patients were treated before the occurrence of proteinuria and impaired renal function (Savige et al., 2013; Torra and Furlano, 2019). So early detection of ADAS patients with risk to ESRD is extremely important which could be greatly diagnosed with the help of next-generation and whole-exome sequencing methods. Unfortunately, the cost of the technique is still quite expensive and health insurance cannot cover the cost in China.

In conclusion, the presented case demonstrates how NGS in the combination of bioinformatics analysis strategy, together with clinical, histologic, and pedigree information, provides a specific diagnosis in patients with a family history of kidney disease and illustrates different phenotypes of ADAS family members with the same genotype. More importantly, a novel heterogenous missense *COL4A3* mutation, c.3566G > A (p.G1189E), was identified. Our study showed the importance of application of NGS for early ADAS diagnosis which may potentially improve the prognosis of ADAS population. It also expands the spectrum of *COL4A3* mutations, helps illustrate genotype–phenotype correlation, and contributes to genetic diagnosis and counseling of Alport syndrome.

## Data Availability

The datasets for this article are not publicly available due to concerns regarding participant/patient anonymity. Requests to access the datasets should be directed to the corresponding authors.
