# Supplementary material for: Case Report: Identification of a Novel Heterozygous Missense Mutation in COL4A3 Gene Causing Variable Phenotypes in an Autosomal-Dominant Alport Syndrome Family
Source: Front Genet. 2022 Mar 29;13:839212. doi: 10.3389/fgene.2022.839212 (PMC9001967; doi:10.3389/fgene.2022.839212)
Supplement: Supplementary file 1 [file DataSheet1.PDF]

Supplementary materials

Table 1 Candidate gene

Gene	AAChange.refGene	ACMG	1000g2015au	1000g2015aug_eas	ExAC_ALL	ExAC_EAS	gnomAD_exome_Al	gnomAD_	REVEL
**COL4A3**	**COL4A3:NM_000091:exon42:c.G3566A:p.G1189E**	**Likely pathogenic（PM1+PM2+PP3+PP4）**	**0**	**0**	**0**	**0**	**0**	**0**	**0.974**
NPHS1	NPHS1:NM_004646:exon23:c.G3130A:p.E1044K	Uncertain significance（PM2）	0	0	0.0001	0.0017	0.0001	0.0011	0.065
NPHS1	NPHS1:NM_004646:exon2:c.G115A:p.E39K	Uncertain significance（PM1+PM2+BP4）	0	0	0.0001	0.0016	0.0001	0.001	0.054
FAT1	FAT1:NM_005245:exon10:c.C8087T:p.P2696L	Uncertain significance（PM1+PM2）	0.00019968	0	9.94E-05	0	8.94E-05	5.80E-05	0.4

Table 2 Clinical presentation and diagnosis associated with candidate genes

Gene	HGMD clinical presentation
**COL4A3**	**Microhaematuria_and_proteinuria, Alport_syndrome_/_thin_basement_membrane_nephropathy, Alport_syndrome, Collagen_IV_nephropathy, Glomerular_kidney_disease, Benign_haematuria, Proteinuria_and_hematuria, Focal_segmental_glomerulosclerosis, Deafness, Steroid-resistant_nephrotic_syndrome_with_mesangial_proliferation, Chronic_obstructive_pulmonary_disease_association_with, Glomerular_microscopic_haematuria, Haematuria, Glomerulonephritis, Alport_syndrome_autosomal_dominant, Nephrotic_syndrome_steroid_resistant, Left_ventricular_obstruction, Nephronophthisis, Phenotype_modifier, Nephrotic_syndrome_monogenic**
NPHS1	Nephrotic_syndrome_steroid_resistant, Nephrotic_syndrome, Nephrotic_syndrome_monogenic, Focal_segmental_glomerulosclerosis, Congenital_nephrotic_syndrome_Finnish_type, Diffuse_mesangial_sclerosis, Proteinuria_and_hematuria, Glaucoma_ASD_dysmorphic_facial_features_nephrotic_syndrome_developmental_delay_short_stature_&_feeding_difficulties, Glomerular_kidney_disease, Minimal_change_nephrotic_syndrome, Refractory_lupus_nephritis
NPHS1	Nephrotic_syndrome_steroid_resistant, Nephrotic_syndrome, Nephrotic_syndrome_monogenic, Focal_segmental_glomerulosclerosis, Congenital_nephrotic_syndrome_Finnish_type, Diffuse_mesangial_sclerosis, Proteinuria_and_hematuria, Glaucoma_ASD_dysmorphic_facial_features_nephrotic_syndrome_developmental_delay_short_stature_&_feeding_difficulties, Glomerular_kidney_disease, Minimal_change_nephrotic_syndrome, Refractory_lupus_nephritis
FAT1	Congenital_anomalies_of_the_kidney_and_urinary_tract, Facioscapulohumeral_dystrophy-like_phenotype, Spinocerebellar_ataxia, Autism_spectrum_disorder, Sarcoidosis, Autism, Nephrotic_syndrome_tubular_ectasia_and_haematuria, Acinar_cell_carcinoma_of_the_pancreas

Table 3 genetic kidney disease related genes

MSH6	SDHD	ABCA1	ABCC6	ABCD3	ABCD4	ACAT1	ACE	ACSF3	ACTN4	ADCK3	ADCK4	ADCY6	ADGRG6	AGL
AGT	AGTR1	AGXT	AHI1	AHNAK	AKT1	AKT3	ALDOA	ALDOB	ALG1	ALG11	ALG12	ALG13	ALG2	ALG3
ALG6	ALG6T	ALG8	ALG9	ALMS1	ALPL	ANKRD1	ANKS6	ANLN	ANOS1	AP3B1	APC	APOA1	APOA2	APOL1
APOPT1	APRT	AQP2	AR	ARHGAP24	ARHGDIA	ARL13B	ARL6	ARMC5	ARNT2	ASL	ASS1	ATP6V0A4	ATP6V1B1	ATP7B
ATRX	AVPR2	AXIN2	B2M	B3GLCT	B4GALT1	B9D1	B9D1	B9D2	BAAT	BARD1	BBIP1	BBS1	BBS10	BBS12
BBS2	BBS4	BBS5	BBS7	BBS9	BCL10	BCS1L	BICC1	BLOC1S3	BLOC1S6	BMP4	BMP7	BMPER	BMPR1A	BRAF
BRCA2	BRIP1	BSND	BUB1	BUB1B	C2CD3	C3	C3AR1	C5orf42	CA2	CAD	CASR	CC2D2A	CCBE1	CCDC115
CCDC28B	CCND1	CCND2	CD2AP	CD320	CD46	CD96	CDC5L	CDC73	CDH1	CDH5	CDKN1B	CDKN1C	CDKN2A	CDKN2A
CDKN2B	CEP104	CEP120	CEP164	CEP290	CEP41	CEP57	CEP83	CFB	CFH	CFHR1	CFHR3	CFHR5	CFI	CHD1L
CHD7	CHEK2	CHRM3	CLCN5	CLCNKA	CLCNKB	CLDN16	CLDN19	CLPB	CNNM2	CNTN5	CNTNAP1	COA3	COA5	COA6
COG1	COG4	COG5	COG6	COG7	COG8	COL4A1	COL4A3	COL4A4	COL4A5	COL4A6	COQ2	COQ4	COQ6	COQ7
COQ9	COX10	COX14	COX15	COX20	COX6B1	COX8A	CPT1A	CPT2	CRB2	CREBBP	CSPP1	CTNNB1	CTNS	CUBN
CUL3	CXCL8	CYP11B1	CYP11B2	CYP21A2	CYP24A1	CYP27B1	DACH1	DCDC2	DCN	DGKE	DHCR7	DHODH	DIRC2	DIRC3
DIS3L2	DKC1	DMP1	DNM2	DOLK	DPAGT1	DPM1	DPM2	DRD1	DSTYK	DTNBP1	DVL1	DVL3	DYNC2H1	EDN3
EDNRA	EDNRB	EGF	EHHADH	EIF2AK3	EMP2	EMX2	ENO3	ENPP1	EPCAM	EPHA3	EPO	ERBB2	ERBB3	ERBB4
ERCC4	ERCC5	ERCC6	ERCC8	ESCO2	ETFA	ETFB	ETFDH	EVC2	EXOC4	EXOC8	EYA1	FAAP95	FAH	FAM20A
FAM20C	FAM58A	FAN1	FANCA	FANCC	FANCD2	FANCE	FANCF	FANCI	FANCM	FASTKD2	FAT1	FAT3	FAT4	FBN1
FBXW7	FGA	FGF10	FGF20	FGF23	FGF8	FGFR1	FGFR2	FGFR3	FH	FLCN	FLNB	FLT4	FMN1	FN1
FOXC1	FOXC2	FOXI1	FRAS1	FREM1	FREM2	FXYD2	FZD5	G6PC	G6PC3	GAA	GALNT3	GALT	GATA3	GATA6
GBE1	GCDH	GDNF	GEMIN4	GEN1	GLA	GLE1	GLI3	GLIS2	GNAS	GNAS-AS1	GPC3	GRHPR	GRIP1	GSN
GYG1	GYS1	GYS2	H19	HAL	HCCS	HDAC8	HFE	HIF1A	HMGA2	HMGB3	HNF1A	HNF1B	HNF4A	HOGA1
HOXA13	HPRT1	HPS1	HPS3	HPS4	HPS5	HPS6	HPSE2	HRAS	HSD11B2	HSD17B4	HSD3B2	IFNG	IFT122	IFT140
IFT172	IFT27	IFT43	IFT80	IGFBP1	IKBKAP	IL1A	IL1RN	INF2	INPP5E	INSL3	INVS	IQCB1	IRAK3	IRF4
IRF6	ITGA3	ITGA8	ITGB4	JAG1	JAM3	KAL1	KANK1	KANK2	KANK4	KANSL1	KAT6B	KCNA1	KCNIP4	KCNJ1
KCNJ10	KCNJ5	KCNQ1OT1	KDM6A	KDR	KIAA0556	KIAA0586	KIF14	KIF1B	KIF7	KL	KLHL3	KRAS	LAMA2	LAMB2
LDHA	LMBRD1	LMX1B	LPIN1	LRBA, MAB2	LRIG2	LRP10	LRP2	LRP4	LYZ	LZTFL1	LZTR1	MAFB	MAX	MBTPS2
MC2R	MCC	MCM4	MEFV	MEN1	MET	MGAT2	MITF	MKKS	MKS1	MLH1	MLH3	MLL2	MMAA	MMAB
MMACHC	MMADHC	MNX1	MOGS	MPDU1	MPI	MRE11A	MRPL23	MRPS7	MSH2	MSH6	MT-CO1	MT-CO2	MT-CO3	MTHFR
MTOR	MTR	MTRR	MT-TE	MTTL1	MT-TN	MT-TS1	MUC1	MUT	MUTYH	MVK	MYBPC1	MYH9	MYO15A	MYO1E
NAT8	NBN	NEK1	NEK8	NF1	NF2	NGLY1	NIPBL	NLRP3	NME1	NOTCH2	NPC1	NPC2	NPHP1	NPHP3
NPHP4	NPHP5	NPHS1	NPHS2	NR3C2	NR5A1	NRAS	NSD1	NSDHL	NTF3	NUP107	NUP205	NUP93	NXF5	OCLN
OCRL	ODC1	OFD1	OGG1	OSR1	OTX2	PALB2	PAX2	pax2tvc	PAX6	PC	PDE6D	PDE8B	PDGFRL	PDSS1
PDSS2	PEBP1	PECAM1	PET100	PEX1	PEX5	PFKM	PGAM2	PGK1	PGM1	PHEX	PHF9	PHKA1	PHKA2	PHKB
PHKG2	PIGA	PIGL	PIGN	PIGT	PIK3CA	PIK3R2	PIP5K1C	PKD1	PKD2	PKHD1	PLA2G2A	PLA2R1	PLCE1	PLG
PMM2	PODXL	POMC	PON1	POR	PORCN	POU6F2	PPARG	PQBP1	PRCC	PROC	PROK2	PROKR2	PRPS1	PTCH1
PTEN	PTPN11	PTPN12	PTPRJ	PTPRO	PYGL	PYGM	RAB40AL	RAD21	RAD50	RAD51C	RAF1	RAI1	RARB	RBCK1
REN	RET	RFT1	RIT1	RMND1	RNF139	RNU4ATAC	ROBO2	ROCK2	ROR2	RPGRIP1L	RRM2B	RXFP2	SAC	SALL1
SALL4	SARS2	SCARB2	SCNN1A	SCNN1B	SCNN1G	SCNN2G	SCO1	SCO2	SDCCAG8	SDHA	SDHB	SDHC	SDHD	SEMA3D
SEMA3E	SERPINH1	SF3B4	SI	SIX1	SIX2	SIX5	SLC12A1	SLC12A3	SLC13A5	SLC16A12	SLC1A1	SLC22A12	SLC22A5	SLC26A3
SLC2A1	SLC2A2	SLC2A9	SLC34A1	SLC34A3	SLC35A1	SLC35A2	SLC35C1	SLC36A2	SLC37A4	SLC39A8	SLC3A1	SLC4A1	SLC4A4	SLC5A1
SLC5A2	SLC6A19	SLC6A20	SLC7A7	SLC7A9	SLC9A3R1	SLX4	SMAD4	SMARCAL1	SMARCB1	SMC3	SMPD1	SOD2	SOS1	SOS2
SOX17	SOX2	SRCAP	SRD5A3	SRGAP1	SSR4	STK11	STRA6	STT3A	STT3B	STX16	SUCLA2	SURF1	TAT	TBX18
TCTN1	TCTN2	TCTN3	TFAP2A	TFE3	THBD	TINAG	TLR2	TMEM127	TMEM138	TMEM165	TMEM199	TMEM216	TMEM231	TMEM237
TMEM67	TNS3	TNXB	TP53	TP63	TRAP1	TREX1	TRIM32	TRPC6	TRPM6	TSC1	TSC2	TTC21B	TTC8	TWIST1
UBE2QL1	UMOD	UPF3B	UPK3A	VAX1	VDR	VEGFA	VHL	VIPAS39	VPS33B	WDPCP	WDR19	WDR34	WDR35	WDR60
WDR73	WNK1	WNK4	WNT10A	WNT11	WNT3	WNT4	WNT5A	WNT6	WNT7A	WT1	XDH	XPNPEP3	XPO5	XRCC2
XRCC6	XRCC9	XYLT1	XYLT2	ZBTB42	ZHX2	ZMPSTE24	ZNF365	ZNF423	ZNF432					
