# Supplementary material for: Case Report: Identification of a Novel Heterozygous Missense Mutation in COL4A3 Gene Causing Variable Phenotypes in an Autosomal-Dominant Alport Syndrome Family
Source: Front Genet. 2022 Mar 29;13:839212. doi: 10.3389/fgene.2022.839212 (PMC9001967; doi:10.3389/fgene.2022.839212)
Supplement: Supplementary file 2 [file DataSheet2.docx]

Supplementary material: Time line of the medical history of the proband

2011

Proteinuria with UPC 1.0-2.9g/g

Mild intermittent hematuria

Normal Scr

2021

Proteinuria with UPC 3.26g/g

Hematuria + with 70% dysmorphic

Normal Scr

2021

Next generation sequencing showed heterozygous missense mutation in COL4A3 gene (c.G3566A: p.G1189E) in all the affected family members

2018

Renal biopsy showed:

Mild mesangial expansion,

diffuse thin GBM and very focal interstitial foam cells infiltration

ACEI

ACEI/ARB+Traditional Chinese Medicine
